# *Edgeworthia gardneri* (Wall.) Meisn. Ethanolic Extract Attenuates Endothelial Activation and Alleviates Cardiac Ischemia-Reperfusion Injury

**DOI:** 10.3390/molecules29051068

**Published:** 2024-02-29

**Authors:** Xiaoya Lang, Chao Zhong, Lingqing Su, Manman Qin, Yanfei Xie, Dan Shan, Yaru Cui, Min Shi, Min Li, Hexiu Quan, Liang Qiu, Guoyue Zhong, Jun Yu

**Affiliations:** 1 Center for Translational Medicine, Jiangxi University of Chinese Medicine, Nanchang 330004, China; langxiaoya@jxutcm.edu.cn (X.L.); 13606525622@163.com (L.S.); 20142016@jxutcm.edu.cn (M.Q.); 20070945@jxutcm.edu.cn (Y.X.); cuiyaru@jxutcm.edu.cn (Y.C.); 20010381@jxutcm.edu.cn (M.S.); 20101063@jxutcm.edu.cn (H.Q.); 20091057@jxutcm.edu.cn (L.Q.); 2 Department of Cardiovascular Sciences, Center for Metabolic Disease Research, Lewis Katz School of Medicine, Temple University, Philadelphia, PA 19140, USA; tuq93597@temple.edu; 3 Center for Traditional Chinese Medicine Resources and Ethnic Medicine, Jiangxi University of Chinese Medicine, Nanchang 330004, China; 20131004@jxutcm.edu.cn (M.L.); 20111025@jxutcm.edu.cn (G.Z.)

**Keywords:** *Edgeworthia gardneri* (Wall.) Meisn., endothelium activation, inflammatory response, cardiac ischemia–reperfusion, mitogen-activated protein kinase

## Abstract

Endothelial pro-inflammatory activation is pivotal in cardiac ischemia–reperfusion (I/R) injury pathophysiology. The dried flower bud of *Edgeworthia gardneri* (Wall.) Meisn. (EG) is a commonly utilized traditional Tibetan medicine. However, its role in regulating endothelium activation and cardiac I/R injury has not been investigated. Herein, we showed that the administration of EG ethanolic extract exhibited a potent therapeutic efficacy in ameliorating cardiac endothelial inflammation (*p* < 0.05) and thereby protecting against myocardial I/R injury in rats (*p* < 0.001). In line with the in vivo findings, the EG extract suppressed endothelial pro-inflammatory activation in vitro by downregulating the expression of pro-inflammatory mediators (*p* < 0.05) and diminishing monocytes’ firm adhesion to endothelial cells (ECs) (*p* < 0.01). Mechanistically, we showed that EG extract inhibited the nuclear factor kappa-B (NF-κB), c-Jun *N*-terminal kinase (JNK), extracellular regulated protein kinase (ERK), and p38 mitogen-activated protein kinase (MAPK) signaling pathways to attenuate EC-mediated inflammation (*p* < 0.05). Collectively, for the first time, this study demonstrated the therapeutic potential of EG ethanolic extract in alleviating I/R-induced inflammation and the resulting cardiac injury through its inhibitory role in regulating endothelium activation.

## 1. Introduction

Coronary artery disease (CAD) is one of the major causes of morbidity and mortality around the world [1]. The primary therapeutic strategy for attenuating acute coronary syndrome is timely coronary artery bypass grafting or intervention [2]. However, restoring blood flow to the ischemic heart causes additional irreversible damage to cardiomyocytes, exacerbating heart tissue damage known as cardiac ischemia–reperfusion (I/R) injury [2]. Upon ischemia, the supply of oxygen and nutrition to the heart is blocked, which persists during the process of reperfusion because of the no-reflow phenomenon, leading to disrupted myocardial energy metabolism [3]. This energy metabolism disorder further facilitates pathological processes, including vascular hyperpermeability, oxidative stress, inflammatory response, cardiomyocyte apoptosis, and excessive autophagy, which synergistically drive the initiation and progression of cardiac I/R injury [3]. Despite remarkable advances in managing cardiac I/R injury during the past decades, the burden of CAD remains high [3]. Therefore, exploring new effective strategies for managing cardiac I/R injury is of great clinical significance.

Endothelium activation and subsequent inflammation have been implicated in the pathophysiology of cardiac I/R injury [4]. Upon I/R insult, robust inflammatory responses are triggered in the myocardium, characterized by the activation of immune signaling cascades, the expression of a large panel of pro-inflammatory mediators, and the recruitment of inflammatory cells [5]. Although adequately orchestrated inflammatory responses are required for the cardiac healing and reparative processes, excessive inflammatory cascades drive the cardiac repair to injury, causing increased myocardial infarction (MI) size followed by adverse cardiac remodeling and the development of heart failure [6]. Notably, it has been reported that targeting the inflammatory response upon cardiac reperfusion results in significant cardioprotective effects [7,8,9,10], suggesting anti-inflammation as a promising strategy for cardiac I/R injury prevention and management. Endothelial cells (ECs), which comprise a major cell population in addition to cardiomyocyte cells in the cardiac tissue, act as crucial protagonists in myocardial homeostasis and disease [11]. As a barrier to inflammation, it has been well established that endothelial pro-inflammatory activation critically mediates the pathogenesis of cardiac I/R injury by generating and releasing a cluster of pro-inflammatory mediators, including adhesion molecules (e.g., vascular cell adhesion molecule-1 [VCAM-1] and intercellular adhesion molecule-1 [ICAM-1]), cytokines (e.g., tumor necrosis factor-α [TNF-α], interleukin-1β [IL-1β] and interleukin-6 [IL-6]), and chemokines (e.g., monocyte chemotactic protein-1 [MCP-1]), thus promoting the recruitment of inflammatory cells and potentiating myocardial inflammatory responses [12]. Inhibiting endothelial inflammation by using antibodies against adhesion molecules significantly reduced cardiac I/R injury [7,8,9]. Therefore, targeting EC-mediated inflammation is expected to exert a beneficial effect on cardiac I/R injury.

The flower bud of *Edgeworthia gardneri* (Wall.) Meisn. (EG) has a long history of medicinal application in traditional Tibetan medicine. People in Tibet commonly use EG to prepare herbal tea to prevent and treat metabolic disorders [13]. EG extracts obtained by different isolation and purification methods have been demonstrated to possess various pharmacological properties, including beneficial effects on diabetes mellitus, insulin resistance, hyperlipidemia, and adipogenesis [14,15,16,17,18]. These biological effects were mediated by various molecular targets, intracellular signaling pathways, and gut microbiota [14,15,16,17,18]. Our recent studies have identified an active non-petroleum ether-soluble fraction of EG as a potential therapeutic agent against atherosclerosis and MI [19,20], highlighting its clinical use in managing cardiovascular disease. However, the effects of the ethanolic extract of EG, which is prepared using 60% ethanol, on endothelium activation and myocardial I/R injury are still unclear. Therefore, the present study aimed to investigate whether EG extract modulates endothelial inflammation and protects against cardiac I/R injury and, if so, through which associated mechanism. Our current study demonstrated that EG ethanolic extract inhibited endothelium activation through diminishing the nuclear factor kappa-B (NF-κB), c-Jun *N*-terminal kinase (JNK), extracellular regulated protein kinase (ERK), and p38 mitogen-activated protein kinase (MAPK) signaling pathways, which consequently attenuated cardiac inflammation and heart infarct size after I/R. 

## 2. Results

### 2.1. Identification of Chemical Constituents in EG Extract

Electrospray ionization (ESI)– mass spectrometry (MS) was carried out to identify the organic compounds in the EG extract. Supplementary Figure S1 shows the total ion chromatogram profiles of the chemical constituents, and Table 1 and Supplementary Figure S2 display the detailed information and the chemical structures of the identified compounds. Twenty-eight compounds were found in the EG extract (Table 1), which can be classified into several groups of naturally occurring molecules, as follows.

- Fatty acids and their derivatives: ethyllinolenate (**1**), tridecanoic acid (**2**), pentadecanoic acid (**3**), and hexadecanoic acid (**24**).
- Flavonoids: kaempferol (**4**), astragalin (**5**), apigenin (**6**), daidzein (**7**), and kaempferol 3-*O*-rutinoside (**25**).
- Polypeptides: Pro-lle (**8**), GIn-Asp (**9**), Cys (Trioxidation)-Pro (**10**), and Glu-His (**11**).
- Coumarins: daphnoretin (**12**), and dihydrokaempferol (**13**).
- Amino acid: dl-arginine (**14**).
- Aldehyde: palmital (**15**).
- Phenylpropanoid: femlic acid (**26**).
- Other compounds: adenosine (**16**), xanthene-9-carboxylic acid (**17**), mevalonic acid (**18**), 1,4-benzenediol, 2-methyl-(**19**), sebacic acid (**20**), jasmonic acid (**21**), salicylic acid (**22**), dimethyl phthalate (**23**), 9*S*,11*R*,15*S*-trihydroxy-5Z-prostenoic acid (**27**), and 3-hydroxy-3-methylglutaric acid (**28**).

### 2.2. EG Extract Attenuates Cardiac I/R Injury In Vivo

**Figure 1:**
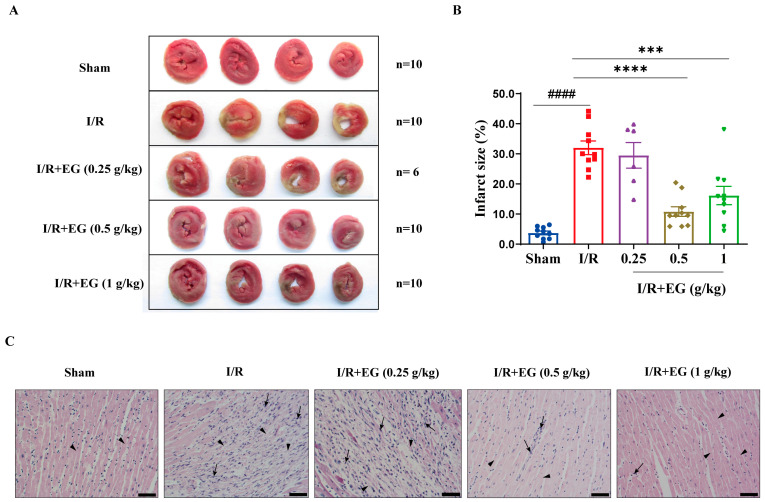
EG extract improves infarct size and tissue structural pathological changes upon cardiac I/R injury. (**A**) Representative TTC staining of myocardial tissue 48 h after cardiac I/R or sham surgery from rats administrated with vehicle or EG extract (0.25, 0.5, and 1 g/kg). The white region reflects infarct zoon. (**B**) Quantification of infarct area as a percentage of total slice area as indicated by TTC staining using vehicle- or EG extract (0.25, 0.5, and 1 g/kg)-treated hearts that underwent cardiac I/R or sham operation (*n* = 6–10). (**C**) Representative H&E staining of the vehicle- or EG extract (0.25, 0.5, and 1 g/kg)-treated cardiac tissues 48 h post cardiac I/R or sham operation (scale bar, 50 μm). Arrowheads indicate myocardial fibers, and arrows indicate inflammatory cell infiltration. Data are mean ± SEM. *p* values were calculated by one-way ANOVA followed by Tukey’s post hoc test. ^####^ *p* < 0.0001, *** *p* < 0.001, **** *p* < 0.0001.

To investigate the potential cardioprotective effect of EG, rats treated with EG extract or vehicle were subjected to cardiac I/R or sham surgery. Subsequently, TTC staining was performed to analyze the infarct size to determine cardiac I/R-induced necrosis. As expected, vehicle-treated rat hearts that underwent 48 h of cardiac I/R exhibited a significantly increased infarct size, as indicated by the white area in myocardial tissue compared to sham-operated controls (Figure 1A,B). However, the infarct size of EG extract (0.5 and 1 g/kg)-treated hearts was markedly decreased compared with vehicle controls at 48 h after cardiac I/R (Figure 1A,B). These data demonstrate that EG extract alleviates cardiac I/R-induced infarction. To assess the pharmacological action of EG extract on the morphology and structure of I/R-challenged myocardium, H&E staining was conducted using sham- and I/R-operated hearts from vehicle- and EG extract-treated rats at 48 h after surgery. As shown in Figure 1C, the cardiomyocytes were arranged regularly in sham-operated controls without inflammatory cell accumulation. In contrast, I/R-operated hearts exhibited disordered myocardial fiber arrangement (arrow heads), extensive deposition of extracellular matrix, and overwhelming inflammatory cell infiltration (arrows). Notably, EG extract administration significantly relieved these damages, manifesting improved myocardial fiber structure and attenuated infiltration of inflammatory cells (Figure 1C). These results suggest that EG extract ameliorates cardiac histopathological damage in response to I/R insult. Taken together, EG extract exerts a protective effect on cardiac I/R injury in rats.

### 2.3. EG Extract Suppresses I/R-Evoked Cardiac Inflammation In Vivo

**Figure 2:**
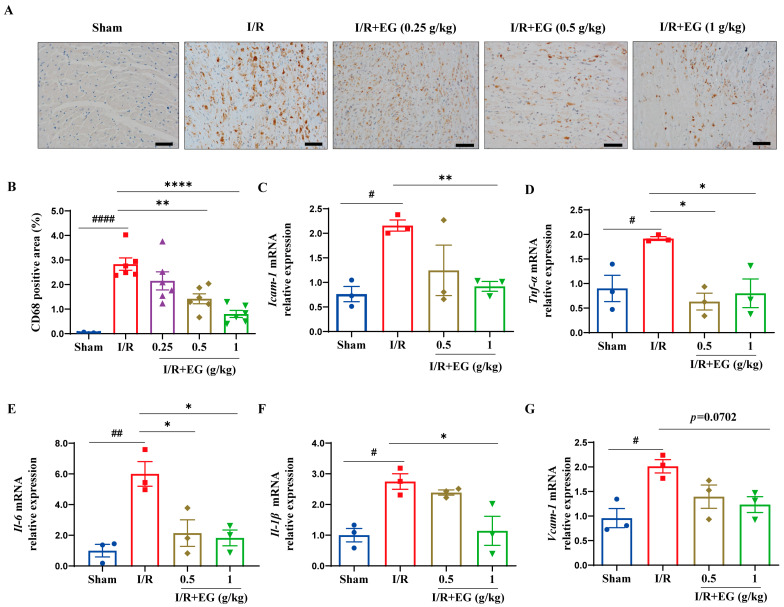
EG extract inhibits cardiac inflammation in response to I/R insult. (**A**) Representative images of CD68 immunohistochemical staining using hearts obtained from vehicle- or EG extract (0.25, 0.5, and 1 g/kg)-treated rats 48 h after cardiac I/R or sham operation (scale bar, 50 μm). (**B**) Quantification of CD68-positive areas as indicated by immunohistochemical staining using hearts 48 h after cardiac I/R or sham operation from the vehicle- or EG extract (0.25, 0.5, and 1 g/kg)-treated rats (*n* = 6). (**C**–**G**) qRT-PCR analysis of genes associated with inflammation *Icam-1* (**C**), *Tnf-α* (**D**), *Il-6* (**E**), *Il-1β* (**F**), and *Vcam-1* (**G**) were performed using mRNA extracted from the vehicle- or EG extract (0.5 and 1 g/kg)-treated hearts 48 h post cardiac I/R or sham surgery (*n* = 3). Data are mean ± SEM. *p* values were calculated by one-way ANOVA followed by Tukey’s post hoc test. ^#^ *p* < 0.05, ^##^ *p* < 0.01, ^####^ *p* < 0.0001, * *p* < 0.05, ** *p* < 0.01, **** *p* < 0.0001.

Induction of inflammatory responses, including the expression of pro-inflammatory mediators and the recruitment of inflammatory cells, is mechanistically implicated in the pathophysiology of myocardial I/R injury [21]. Importantly, excessive inflammatory responses cause detrimental effects on the heart tissue by impairing cardiac healing and aggravating adverse cardiac remodeling after I/R insult [22]. To assess the impact of EG extract on cardiac inflammation following I/R, the recruitment and infiltration of CD68^+^ macrophages into the myocardium were examined using immunohistochemical staining. Cardiac I/R surgery markedly increased the number of CD68^+^ macrophages compared to sham-operated rats (Figure 2A,B). Nevertheless, EG extract treatment (0.5 and 1 g/kg) decreased the recruitment and infiltration of CD68^+^ macrophages compared to the vehicle controls at 48 h after cardiac I/R (Figure 2A,B). To further evaluate the potential anti-inflammatory effect of EG extract, the expression levels of pro-inflammatory mediators were examined in hearts from vehicle- and EG extract-treated rats that underwent sham or cardiac I/R surgery. As shown in Figure 2C–G, the expression of inflammation-related genes *Icam-1*, *Vcam-1*, *Tnf-α, Il-6*, and *Il-1β* in the vehicle-treated left ventricle was significantly upregulated at 48 h after cardiac I/R, compared with those from sham-operated controls. These effects were observed to be profoundly reversed by EG extract treatment (Figure 2C–G). Notably, adhesion molecules *Icam-1* and *Vcam-1*, two well-known indicators of endothelium activation that mediate tight adherence of inflammatory cells to ECs, were downregulated by EG extract (Figure 2C–G), suggesting that EG extract ameliorates endothelial pro-inflammatory activation during cardiac I/R injury. Collectively, these findings demonstrate that EG extract attenuates cardiac inflammatory responses following I/R, which may benefit myocardial I/R injury in vivo.

### 2.4. EG Extract Inhibits the Inflammatory Responses in Activated ECs

**Figure 3:**
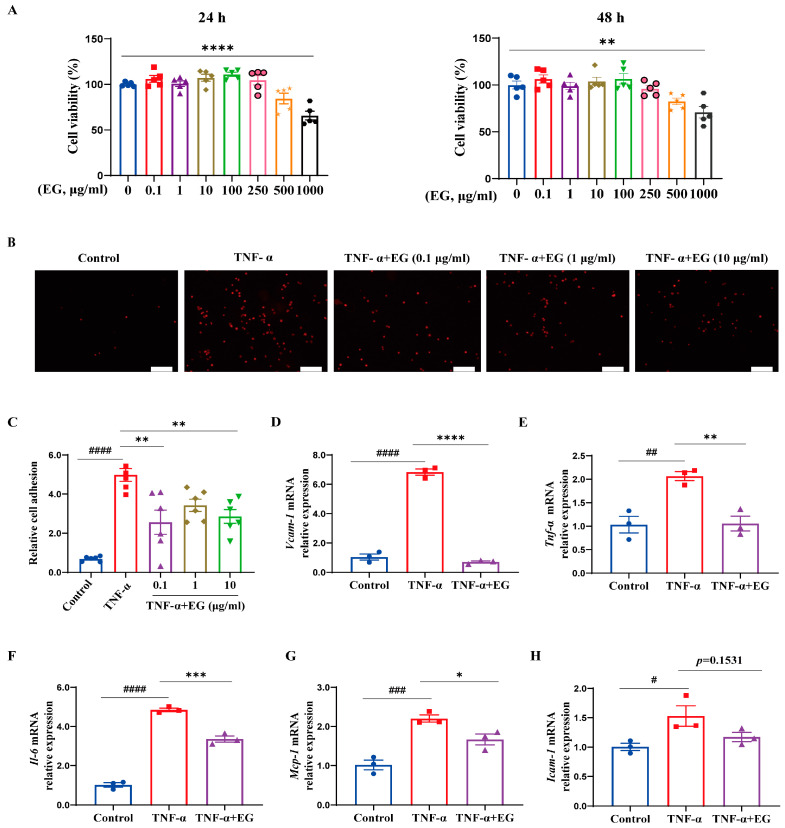
EG extract suppresses the inflammatory responses in TNF-α-induced HUVECs. (**A**) HUVECs were grown to reach confluency and then treated with different doses of EG extract. MTT assays measured cell viability at 24 and 48 h (*n* = 5). (**B**) Representative images showing THP-1 monocytes’ adhesion to TNF-α-induced HUVECs pretreated with or without EG extract (0.1–10 μg/mL) (scale bar, 100 μm). (**C**) Quantitative analysis of the adherent THP-1 monocytes (*n* = 6). (**D**–**H**) qRT-PCR analysis of *VCAM-1* (**D**), *TNF-α* (**E**), *IL-6* (**F**), *MCP-1* (**G**), and *ICAM-1* (**H**) in TNF-α-induced HUVECs pretreated with or without EG extract (200 μg/mL) (*n* = 3). Data are mean ± SEM. *p* values were calculated by one-way ANOVA followed by Tukey’s post hoc test. ^#^ *p* < 0.05, ^##^ *p* < 0.01, ^###^ *p* < 0.001, ^####^ *p* < 0.0001, * *p* < 0.05, ** *p* < 0.01, *** *p* < 0.001, **** *p* < 0.0001.

Endothelial pro-inflammatory activation has been recognized as crucial in mediating I/R-induced cardiac inflammation and related heart injury [22]. Activated ECs in response to myocardial I/R express a wide array of inflammatory factors, including adhesion molecules, pro-inflammatory cytokines, and chemokines [12]. These pro-inflammatory mediators promote the recruitment of multitudinous immune cells to the injured heart, contributing to the progress and deterioration of the cardiac pathological process [12]. Thus, we postulate that modulation of endothelial inflammation is involved in EG’s anti-inflammatory property, as observed in Figure 2. To test this hypothesis, we used the TNF-α-induced HUVECs as an in vitro model to determine the influence of EG extract on endothelium activation. First, an MTT assay was performed to examine the effect of EG extract on HUVEC viability. The result showed that HUVECs exposed to 0.1–250 μg/mL of EG extract exhibited no apparent cell cytotoxicity (Figure 3A). Since endothelial pro-inflammatory activation is characterized by the leukocyte–EC adhesion event [23], we then explored the effect of EG extract on firm adhesion of THP-1 monocytes to TNF-α-induced HUVECs. PKH26-labeled THP-1 cells showing red fluorescence were incubated with a TNF-α-induced single layer of confluent HUVECs treated with or without EG extract. After removing non-adherent cells, red fluorescence-labeled adhering THP-1 cells were quantified. As shown in Figure 3B,C, we observed a robust increase in adherence of THP-1 monocytes to HUVECs in response to TNF-α stimulation, which was significantly repressed by the pretreatment with EG extract. Furthermore, we tested whether EG extract could reduce inflammation-related gene expression in TNF-α-stimulated HUVECs. We found a pronounced increase in the expression of pro-inflammatory mediators *VCAM-1*, *ICAM-1*, *TNF-α*, *IL-6*, and *MCP-1* in TNF-α-treated HUVECs (Figure 3D–H). However, EG extract treatment prominently attenuated these responses to TNF-α stimulation (Figure 3D–H). Together with the in vivo anti-inflammatory studies of EG extract (Figure 2), these data support a mechanism by which EG extract attenuates EC pro-inflammatory activation, thereby downregulating cardiac inflammation, which subsequently plays a protective role in alleviating cardiac I/R injury.

### 2.5. EG Extract Diminishes EC-Mediated Inflammation by Dampening NF-κB and MAPK Activation

**Figure 4:**
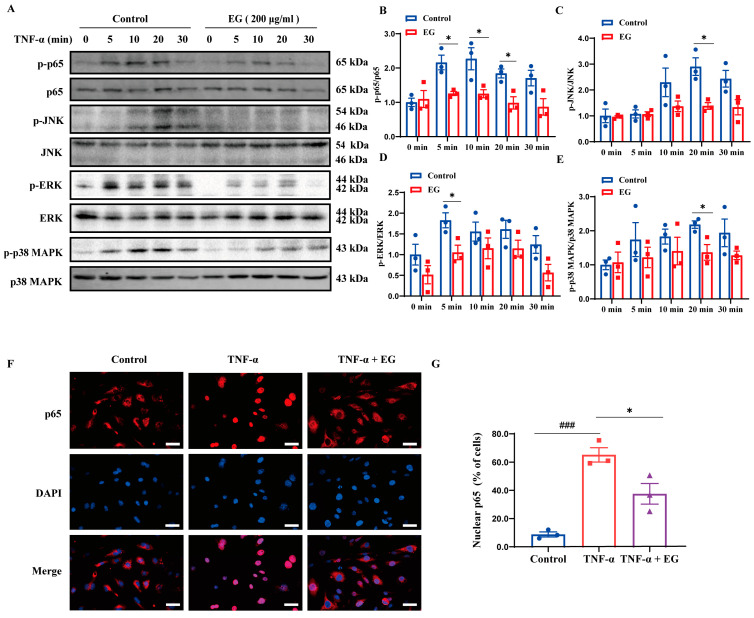
EG extract attenuates NF-κB and MAPK signaling pathways in TNF-α-stimulated HUVECs. (**A**) Immunoblotting assay of p65, JNK, ERK, and p38 MAPK phosphorylation in the vehicle- or EG extract (200 μg/mL)-treated HUVECs in response to TNF-α (10 ng/mL) stimulation for 0, 5, 10, 20, and 30 min. (**B**–**E**) Quantification of p-p65/p65 (**B**), p-JNK/JNK (**C**), p-ERK/ERK (**D**), and p-p38 MAPK/p38 MAPK (**E**) relative expression (*n* = 3). (**F**) Representative immunofluorescence images of p65 nuclear translocation using vehicle- or EG extract (200 μg/mL)-treated HUVECs in response to TNF-α (10 ng/mL) stimulation for 13 min (scale bar, 25 μm). (**G**) Quantification of the percentage of nuclear p65-positive cells (*n* = 3). Data are mean ± SEM. *p* values were calculated by a two-tailed Student’s *t*-test. ^###^ *p* < 0.001, * *p* < 0.05.

It has been well documented that NF-κB and MAPKs (JNK, ERK, and p38 MAPK) act as crucial mediators of endothelium activation by triggering a series of inflammation-related signaling cascades in ECs [23,24]. To determine whether EG extract affects these inflammatory signaling pathways, we examined the phosphorylated protein levels of NF-κB/p65 and MAPKs in TNF-α-stimulated HUVECs treated with or without EG extract. As shown in Figure 4A–E, we found a significant upregulation of NF-κB/p65, JNK, ERK, and p38 MAPK phosphorylation in vehicle-treated HUVECs upon TNF-α stimulation. However, EG extract-treated HUVECs exhibited substantially lower phosphorylation of these proteins (Figure 4A–E). Additionally, the immunofluorescence assay showed that EG extracts robustly mitigated nuclear translocation of NF-κB/p65 in HUVECs in response to TNF-α stimulation (Figure 4F,G). These results demonstrate that EG extract decreases NF-κB and MAPK activation in TNF-α-induced ECs.

**Figure 5:**
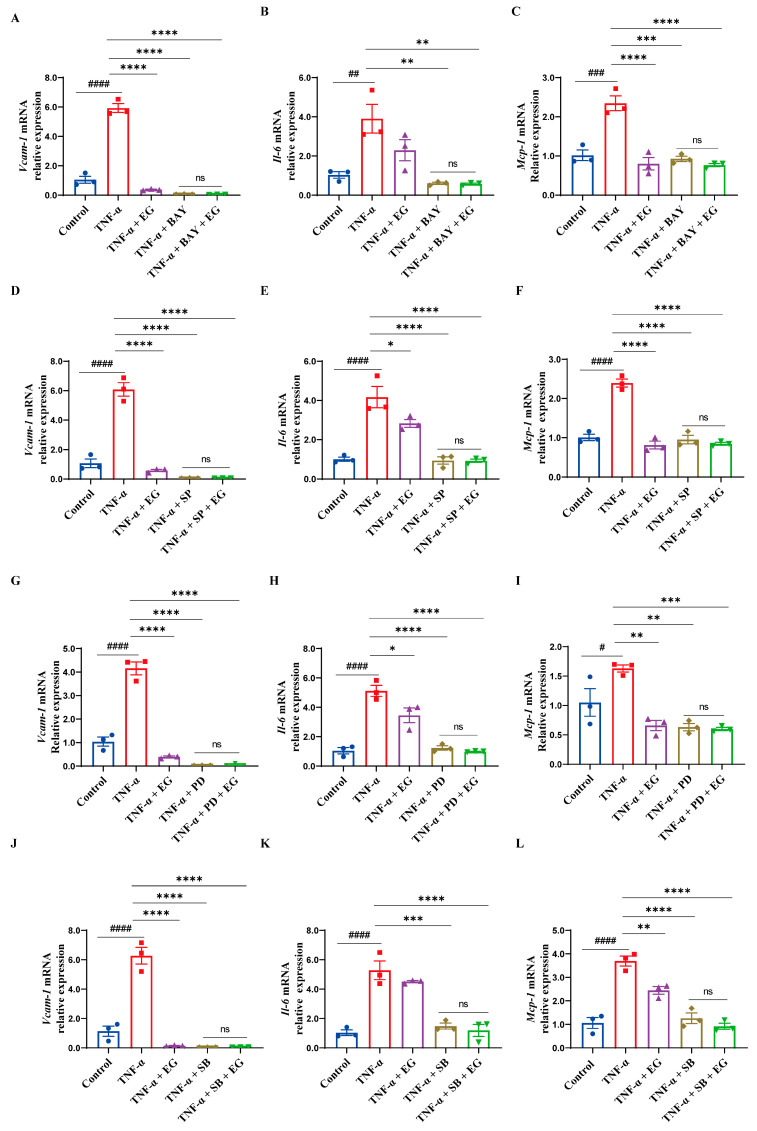
The inhibitory effect of EG extract on endothelial pro-inflammatory activation can be blocked by NF-κB and MAPK inhibitors. (**A**–**C**) qRT-PCR analysis of *VCAM-1*, *IL-6*, and *MCP-1* in TNF-α-induced HUVECs pretreated with vehicle or EG extract (200 μg/mL) while in the absence or presence of BAY11-7082 (BAY, NF-κB inhibitor, 5 μM) (*n* = 3). (**D**–**F**) qRT-PCR analysis of *VCAM-1*, *IL-6*, and *MCP-1* in TNF-α-induced HUVECs pretreated with vehicle or EG extract (200 μg/mL) while in the absence or presence of SP600125 (SP, JNK inhibitor, 20 μM) (*n* = 3). (**G**–**I**) qRT-PCR analysis of *VCAM-1*, *IL-6*, and *MCP-1* in TNF-α-induced HUVECs pretreated with vehicle or EG extract (200 μg/mL) while in the absence or presence of PD98059 (PD, ERK inhibitor, 50 μM) (*n* = 3). (**J**–**L**) qRT-PCR analysis of *VCAM-1*, *IL-6*, and *MCP-1* in TNF-α-induced HUVECs pretreated with vehicle or EG extract (200 μg/mL) while in the absence or presence of SB203580 (SB, p38 MAPK inhibitor, 20 μM) (*n* = 3). Data are mean ± SEM. *p* values were calculated by one-way ANOVA followed by Tukey’s post hoc test. ^#^ *p* < 0.05, ^##^ *p* < 0.01, ^###^ *p* < 0.001, ^####^ *p* < 0.0001, * *p* < 0.05, ** *p* < 0.01, *** *p* < 0.001, **** *p* < 0.0001. “ns” indicates no significance.

**Figure 6:**
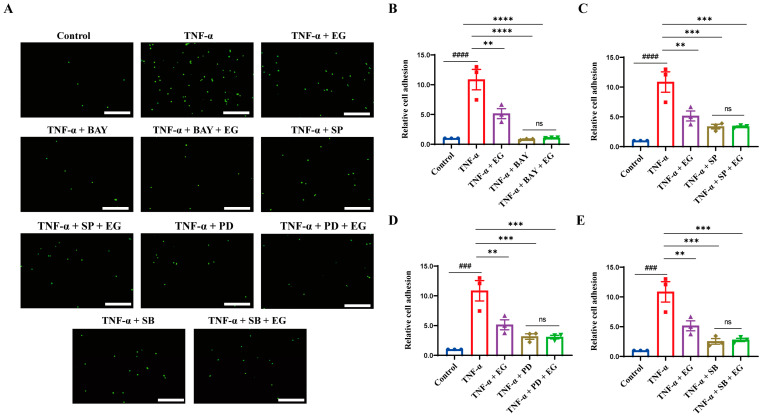
The inhibitory effect of EG extract on firm adhesion of THP-1 monocytes to TNF-α-induced HUVECs can be blocked by NF-κB and MAPK inhibitors. (**A**) Representative images showing THP-1 monocytes adhesion to TNF-α-induced HUVECs pretreated with or without EG extract (200 μg/mL) while in the absence or presence of BAY11-7082 (BAY, NF-κB inhibitor, 5 μM), SP600125 (SP, JNK inhibitor, 20 μM), PD98059 (PD, ERK inhibitor, 50 μM), or SB203580 (SB, p38 MAPK inhibitor, 20 μM) (scale bar, 100 μm). (**B**–**E**) Quantitative analysis of the adherent THP-1 monocytes (*n* = 3). Data are mean ± SEM. *p* values were calculated by one-way ANOVA followed by Tukey’s post hoc test. ^###^ *p* < 0.001, ^####^ *p* < 0.0001, ** *p* < 0.01, *** *p* < 0.001, **** *p* < 0.0001. “ns” indicates no significance.

To further determine that the modulation of NF-κB/MAPK signaling is required for diminished endothelial pro-inflammatory activation mediated by EG extract, we used specific pharmacological inhibitors BAY11-7082 (NF-κB inhibitor), SP600125 (JNK inhibitor), PD98059 (ERK inhibitor), or SB203580 (p38 MAPK inhibitor), and then compared the effect of EG extract on inflammation-related gene expression in TNF-α-induced HUVECs in the absence or presence of inhibitors mentioned above. The results showed that BAY11-7082 or BAY11-7082+EG treatment significantly inhibited the expression levels of pro-inflammatory mediators *VCAM-1*, *IL-6*, and *MCP-1* induced by TNF-α (Figure 5A–C). Notably, the expression of these inflammation-related genes displayed no statistically differences between BAY11-7082 and BAY11-7082+EG treatment groups (Figure 5A–C). Similarly, pro-inflammatory mediator gene expression (*VCAM-1*, *IL-6*, and *MCP-1*) was also markedly reduced by SP600125, PD98059, or SB203580, and the combination of EG extract with each of these three inhibitors did not exert an additive or synergistic effect on the expression of these inflammation-related genes (Figure 5D–L). Consistently, we also observed that the inhibitory effect of EG extract on monocytes’ firm adhesion to HUVECs was blocked by these NF-κB and MAPK inhibitors (Figure 6A–E). Therefore, these findings indicate that EG extract diminishes EC-mediated inflammation by dampening the NF-κB and MAPK (JNK, ERK, and p38 MAPK) signaling pathways.

Taken together, our data suggest that EG extract targets NF-κB and MAPK signaling pathways to attenuate EC-mediated inflammation, alleviating cardiac inflammatory responses and potentially protecting against myocardial I/R injury.

## 3. Discussion

**Figure 7:**
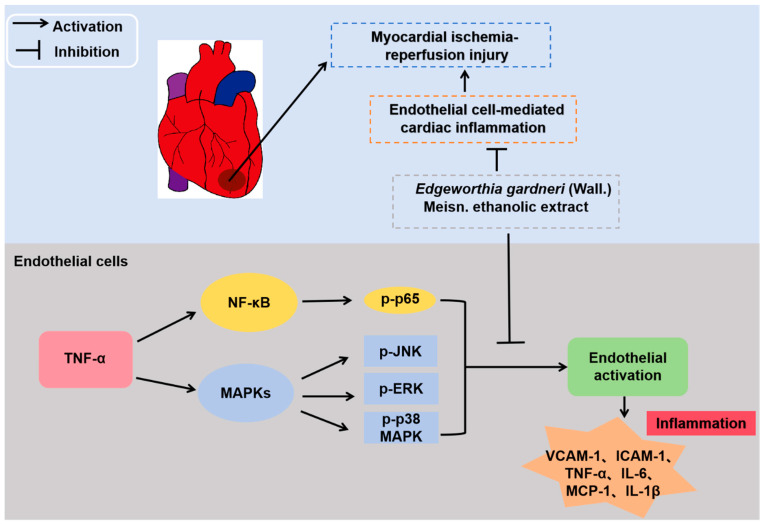
A schematic diagram illustrates EG ethanolic extract’s role in regulating endothelial activation and cardiac I/R injury. EG ethanolic extract mitigates endothelial activation through attenuating NF-κB, JNK, ERK, and p38 MAPK signaling pathways, contributing to the inhibitory effect on cardiac inflammation and, thus, alleviating myocardial I/R injury.

EG is a traditional Chinese herbal medicine with various pharmacological properties [14,15,16,17,18]. However, whether the ethanolic extract of EG regulates endothelium activation, which has been implicated in cardiac I/R injury, is still unclear, a major challenge in treating ischemic heart disease. In this study, we demonstrated that EG extract attenuates endothelial pro-inflammatory activation and inhibits cardiac inflammation, thereby protecting against myocardial I/R injury in rats. Mechanistically, the pharmacological action of EG extract occurs through mitigating the activation of NF-κB and MAPKs in ECs. Our current study demonstrated the therapeutic potential of EG ethanolic extract in combating I/R-induced inflammation and its related cardiac injury (Figure 7).

Inflammation, which involves the release of damage-associated molecular patterns, the induction of inflammatory signaling cascades, the production of inflammation-associated factors, and the recruitment and infiltration of inflammatory cells, has been recognized as central to the pathophysiology of cardiac I/R injury [25,26]. Accordingly, therapeutic targeting of these inflammatory processes holds great promise for treating cardiac I/R injury [27]. In this study, we showed that EG ethanolic extract protects I/R-challenged rat myocardium, manifesting diminished infarct size and improved myocardial histopathological damage, indicating the beneficial effects of EG extract on cardiac I/R injury (Figure 1). Importantly, the therapeutic benefit of EG ethanolic extract can be attributed to its ability to attenuate I/R-induced excessive cardiac inflammation, as indicated by the reduced recruitment of CD68^+^ macrophages and the decreased expression of pro-inflammatory mediators such as *Tnf-α, Il-6*, and *Il-1β* in the heart tissue (Figure 2). Notably, the pro-inflammatory mediators *Icam-1* and *Vcam-1*, two members of the immunoglobulin superfamily of adhesion molecules that are known to be upregulated during endothelial activation and mediate leukocyte adherence to ECs, were markedly repressed by EG extract (Figure 2C,G), suggesting that the modulation of EC-mediated inflammation may be involved in EG’s anti-inflammatory activity in vivo.

ECs have been demonstrated as a major cell type among non-cardiomyocytes in the heart and play pivotal roles in regulating cardiovascular-related physiology and pathology [11]. It has been reported that myocardial I/R induces endothelial dysfunction that further drives cardiac pathological processes [12,28]. In particular, endothelial pro-inflammatory activation, a hallmark of endothelial dysfunction, critically contributes to the pathogenesis of cardiac I/R injury [12,28]. Specifically, activated ECs upregulate the expression of adhesion molecules (e.g., VCAM-1 and ICAM-1) and cytokines/chemokines (e.g., TNF-α, IL-6, IL-1β, MCP-1), promoting the migration, adhesion, and infiltration of inflammatory cells into the injured heart and further potentiating cardiac inflammation that exerts detrimental effects on myocardial tissue [28]. Thus, EC-mediated inflammation represents an attractive target for alleviating myocardial I/R injury [7,8,9]. In the present study, TNF-α-induced HUVECs were thus used as an in vitro model of endothelium activation to examine the effect of EG extract on EC-mediated inflammation. We demonstrated that EG extract inhibits the expression of various pro-inflammatory mediators, including *VCAM-1*, *ICAM-1*, *TNF-α*, *IL-6*, and *MCP-1* in TNF-α-stimulated HUVECs (Figure 3D–H). Moreover, due to the attenuation of the expression of these adhesion molecules and pro-inflammatory mediators, monocyte firm adhesion to ECs was also markedly diminished by EG extract treatment (Figure 3B,C). These data strongly indicate that EG extract suppresses endothelium activation. Consistent with our in vitro observations, we also found that EG-treated rat hearts exhibited decreased inflammatory cell (CD68^+^ macrophages) recruitment and lower expression levels of inflammation-related genes, including *Icam-1*, *Vcam-1*, *Tnf-α, Il-6*, and *Il-1β* in response to I/R insult in vivo (Figure 2). Taken together, our results suggest that EG extract ameliorates endothelium activation, thereby resulting in the attenuation of cardiac inflammation and thus protecting against cardiac I/R injury.

A growing body of evidence has demonstrated that NF-κB and MAPKs are the primary mediators responsible for endothelium activation [29,30] and potential therapeutic targets of cardiac I/R injury [31,32,33,34]. Thus, we used TNF-α-induced HUVECs to ask whether EG extract regulates endothelium activation by targeting these inflammatory signaling pathways. Our current study showed that EG extract significantly inhibited NF-κB and MAPK signaling activation, as reflected by the decreased phosphorylated protein levels of NF-κB/p65, JNK, ERK, and p38 MAPK (Figure 4A–E), and the reduced NF-κB/p65 nuclear translocation (Figure 4F,G). Importantly, using pharmacological inhibitors against NF-κB/MAPKs, we found that compared with NF-κB/MAPK inhibitor alone, the combination of EG extract with each NF-κB/MAPK inhibitor did not have an additive effect on reducing pro-inflammatory mediator gene expression (Figure 5) and monocyte-EC firm adhesion (Figure 6), indicating that the inhibition of NF-κB/MAPK signaling pathways may be one of the mechanisms contributing to EG extract-mediated attenuation of endothelium activation. Together with the in vivo pharmacological studies of EG extract (Figure 1 and Figure 2), we proposed a working model that EG extract diminishes pro-inflammatory activation of ECs through attenuating NF-κB and MAPK signaling pathways, leading to the alleviated cardiac inflammation and thereby protecting against cardiac I/R injury.

Through ESI-MS analysis, twenty-eight compounds were identified in EG extract (Table 1), which belong to several groups of naturally occurring molecules, including fatty acids, flavonoids, polypeptides, coumarins, amino acids, aldehydes, and phenylpropanoids. Notably, some of these compounds have been reported to possess anti-endothelial inflammation properties. For example, kaempferol significantly suppressed pro-inflammatory cytokine production in H_2_O_2_-stimulated HUVECs and a mouse model of acute vascular injury, making it a promising candidate for preventing and treating cardiovascular disease [35]. In advanced glycation end product-induced HUVECs, apigenin inhibited the expression of pro-inflammatory cytokines and adhesion molecules, which was mediated by the attenuated ERK/NF-κB signaling pathway [36]. In a TNF-α-stimulated human coronary artery endothelial cell model, salicylic acid mitigated endothelial activation by dampening NF-κB-mediated expression of the pro-inflammatory mediators ICAM-1 and MCP-1 [37]. Similarly, astragalin [38], daidzein [39], and adenosine [40] have also been demonstrated to attenuate the endothelial inflammatory response. In addition, anti-inflammatory activity was observed for daphnoretin [41], dihydrokaempferol [42], jasmonic acid [43], and kaempferol 3-*O*-rutinoside [44], but their modes of action on endothelial activation remain largely unknown. Interestingly, among compounds mentioned above, previous studies have provided evidence that kaempferol [45], astragalin [46], apigenin [47], daidzein [48], adenosine [49], and salicylic acid [50] exerts protective effects on myocardial I/R injury in experimental animals, depending on the anti-inflammation and anti-oxidative stress activities of these natural molecules. Therefore, based on the above studies, it is conceivable that kaempferol, astragalin, apigenin, daidzein, adenosine, and salicylic acid, which show known inhibitory effects on both endothelial activation and cardiac I/R injury, are likely the active compounds responsible for the pharmacological action of EG extract observed in this study. Nevertheless, whether other compounds listed in Table 1 contribute to EG extract’s pharmacological activity is worthy of further investigation.

Although this study focused on the effect of EG extract on EC-mediated inflammation, we cannot exclude that other cell types being cellular inflammatory effectors such as cardiomyocytes, macrophages, neutrophils, lymphocytes, and fibroblasts may also contribute to EG extract-mediated reduction of cardiac inflammation evoked by I/R. Therefore, whether EG extract exerts anti-inflammatory and cardioprotective effects by targeting the above-mentioned cardiac cell types is worthy of further investigation. Furthermore, a spectrum of mechanisms in addition to inflammation are recognized to be involved in the pathophysiology of cardiac I/R injury, including energy metabolism disorder, oxidative stress, apoptosis, and autophagy [51]. Thus, further studies are warranted to determine whether these additional mechanisms are implicated in the protective effect of EG extract on cardiac I/R injury.

It is noteworthy that sex differences play essential roles in determining the pharmacokinetics and pharmacodynamics of drugs. Previous studies have demonstrated that many cardiovascular drugs display sex-biased responses [52], and emerging evidence from clinical trials indicates that the efficacy of various new drugs targeting cardiac I/R injury is associated with potential sex differences [53]. This study showed that EG extract protects against cardiac I/R injury in male rats, but its pharmacological action on female animals is still unclear. In this regard, further pharmacological studies concerning the efficacy, absorption, distribution, metabolism, and excretion of EG extract in males and females are required to develop EG-based pharmacotherapies to manage cardiac I/R injury.

## 4. Materials and Methods

### 4.1. Plant Materials, Chemicals, and Reagents

The flower buds of of *Edgeworthia gardneri* (Wall.) Meisn. were provided and authenticated by Dr. Guoyue Zhong. We have deposited the voucher specimen (GH837) to the College of Traditional Chinese Medicine, Jiangxi University of Chinese Medicine. The chemicals and reagents used in the current study are included in Supplementary Table S1.

### 4.2. Preparation of EG Ethanolic Extract

EG was extracted using the heat reflux method as previously described [19]. Briefly, the EG sample was grounded and macerated with 60% ethanol for 1 h, and then the heat reflux extraction process was conducted (2 h × 3 times) [19]. Subsequently, the resultant solutions were subjected to evaporation with a rotary evaporator and vacuum drying to obtain the EG ethanolic extract. For in vivo studies, EG ethanolic extract was dissolved in saline for animal oral administration. For in vitro experiments, EG ethanolic extract was dissolved in double-distilled water as a stock solution and then freshly diluted with EC growth medium to the final concentration. The extraction yield of EG extract was 20.1% (*w*/*w*).

### 4.3. Electrospray Ionization (ESI)-Mass Spectrometry (MS) Analysis

The chemical constituents in EG ethanolic extract were analyzed by ESI-MS [54] using a linear trap (LTQ) mass spectrometer–coupled electrospray ion source (Thermo Scientific, Waltham, MA, U.S.A.), which was associated with the Xcalibur data-processing system. The spray ionization voltage was 4 kV in positive ion detection mode. The capillary temperature was 200 °C, the capillary voltage was 35 V, and the tube lens voltage of the LTQ-MS was 110 V. The EG extract was dissolved in extractant (ethanol:water = 60%:40%), diluted to 1 ppm solution (used as a spray reagent), and then filtered. A 2 μL/min flow rate was applied to the 1 ppm spray reagent to pass through a quartz capillary tube. The spray solvent became charged reagent droplets in an electric field with a 0.6 MPa nitrogen sheath gas flow, which was followed by the desolventization to generate ions for subsequent MS analysis. The mass spectral scanning range was *m*/*z* 50 to 1000. The width of the parent ion isolation was 1.0–1.2 Da, and the collision energy was 15–40%.

### 4.4. Animals

Male Sprague Dawley rats (8 weeks old, 200 ± 10 g) were purchased from Slack Jingda Laboratory Animal Co., Ltd. (Changsha, China), and the animal license number is SCXK (Xiang) 2013-0004. All rats were housed in a specific pathogen-free environment with controlled standard conditions as previously described [20]. All animal studies were reviewed and approved by the Institutional Animal Care and Use Committee of the Jiangxi University of Chinese Medicine (approval number: JZLLSC20220306025). The study complied with the ARRIVE (Animal Research: Reporting of In Vivo Animal Experiments) guidelines. The sample size of animal experiments according to relevant published studies in the field was chosen, and 6–10 rats per group were used. After 1 week of the acclimatization period, 46 rats were randomly assigned to 5 groups: the sham group (*n* = 10), cardiac I/R surgery group (*n* = 10), cardiac I/R + EG (0.25 g/kg) group (*n* = 6), cardiac I/R + EG (0.5 g/kg) group (*n* = 10), and cardiac I/R + EG (1 g/kg) group (*n* = 10). For rats in the cardiac I/R + EG groups, EG extract was administered orally at a dose of 0.25 g/kg, 0.5 g/kg, and 1 g/kg once daily for 5 days before conducting cardiac I/R surgery. An equal amount of saline was administered simultaneously to the sham- and cardiac I/R-operated groups. The investigators were blinded to the group allocation during euthanizing animals, staining, and assessing outcomes. The exclusion criteria for animals were animal death and the presence of severe clinical alteration of physiological function. No animals/data were excluded from the analyses for all animal studies.

### 4.5. Myocardial I/R Injury Model

Rats were intraperitoneally injected with 1% pentobarbital sodium (60 mg/kg) and were placed supine. Tracheal intubation was then conducted, and the rats were ventilated with room air using an experimental animal breathing apparatus. Subsequently, the rat heart was exposed, and the ligation of the proximal left anterior descending (LAD) coronary artery was carried out with a 6/0 silk suture. The occurrence of ischemia was evaluated based on bleaching of the cardiac tissue as well as elevation of the ST segment in the electrocardiogram. The rats were subjected to ischemia for 1 h, followed by the release of the suture silk, allowing reperfusion to occur. Finally, the thorax was closed, and the rats were allowed to recover on an electric blanket when they had sufficient spontaneous respiration. Sham-operated animals underwent the same procedure without coronary artery ligation. At 48 h after reperfusion, rats were sacrificed, and tissue samples were harvested for subsequent analysis.

### 4.6. Infarct Size Determination

The hearts were collected and rinsed with chilled saline, then frozen at −80 °C for 10 min. Subsequently, heart samples were cut transversally from the apex to the base at 2 mm thick. The slices were next treated with 1% TTC (Solarbio, Beijing, China) at 37 °C for 15 min in the dark, followed by incubation in 10% Neutral Buffered Formalin Fixative (Solarbio) for 24 h. The infarct area appeared white, and the non-infarct myocardial tissue appeared red. Images were taken with a digital camera (Canon, Tokyo, Japan). Myocardial infarct size was quantified as percentage of infarct area over total slice area.

### 4.7. Histological Analysis, Immunohistochemistry, and Immunofluorescence

Cardiac tissues were fixed using 10% Neutral Buffered Formalin Fixative (Solarbio) and subsequently processed for paraffin embedding and sectioned at 5 μm. Sections were then subjected to deparaffinization and hydration using a graded ethanol series. To evaluate the morphology of cardiac tissues, heart sections were stained with hematoxylin and eosin (H&E) (Sigma-Aldrich, St. Louis, MO, USA) as previously described [20]. For immunohistochemistry, following antigen retrieval, endogenous peroxidase of tissue sections was quenched with 3% H_2_O_2_. Sections were then subjected to incubation with primary antibody against anti-CD68 (Abcam, Boston, MA, USA, ab125212) followed by incubation with the horseradish peroxidase-conjugated secondary antibody (Solarbio, SE134). The expression of CD68 was visualized with a 3,3′-Diaminobenzidine (DAB) kit (Cwbio, Taizhou, China) according to the manufacturer’s instructions.

For immunofluorescence staining, cells were fixed in 4% paraformaldehyde, permeabilized with 0.2% Triton X-100, and processed for primary antibody against NF-κB/p65 (Cell Signaling Technology, Danvers, MA, USA, 6956) followed by incubation with the corresponding secondary antibody (Invitrogen, Carlsbad, CA, USA, A-11005). Nuclei were counterstained with DAPI (Solarbio) for 1 min.

### 4.8. Human Umbilical Vein Endothelial Cell (HUVEC) Culture

HUVECs were provided by the Vascular Biology and Treatment Center of Yale University. The HUVECs were cultured in ECM medium (ScienCell, San Diego, CA, USA) supplemented with 10% fetal bovine serum (ScienCell), 1% EC growth factor cocktail (ScienCell), and 1% penicillin/streptomycin (ScienCell) in humidified conditions at 37 °C with 5% CO_2_. The cells at 4 to 10 passages were used for all the experiments.

### 4.9. Cell Viability Assay and Induction of Endothelial Inflammation

HUVEC viability was monitored by MTT assay as previously described [20]. Briefly, HUVECs were seeded at 5 × 10^4^ cells/mL density in 96-well plates and grown to 90% confluence. Cells were subsequently treated with different doses of EG extract as indicated. After 24 h and 48 h, MTT (5 mg/mL, Sigma-Aldrich) incubation was applied at 37 °C for 3–4 h, followed by DMSO (Solarbio) treatment to solubilize the crystal formed within the cells. Absorbances were subsequently measured at 490 nm.

The induction of endothelial inflammation was performed by treatment of HUVECs with 10 ng/mL TNF-α (PeproTech, Cranbury, NJ, USA). To explore the influence of EG extract on endothelium activation, cells were pretreated with EG extract (200 μg/mL) for 24 h, after which TNF-α (10 ng/mL, PeproTech) was used to stimulate cells for 6 h unless otherwise stated.

### 4.10. Monocyte Adhesion Assay

HUVECs were cultured at 1 × 10^5^ cells/well in 24-well plates in the presence or absence of EG extract (0.1–10 μg/mL) for 24 h. The HUVECS were grown to 90% confluence and treated with TNF-α (10 ng/mL, PeproTech) for 6 h. Then, a 1 × 10^6^ human monocyte cell line THP-1 labeled with PKH26 (red fluorescence) or Calcein AM (green fluorescence) was added and incubated for another 2 h at 37 °C. The non-adherent cells were removed, and the numbers of stained adhering cells were counted using a fluorescence microscope (Nikon, Melville, NY, USA).

### 4.11. Quantitative Real-Time PCR (qRT-PCR)

Total RNA was purified from cardiac tissue homogenates or cultured HUVECs using Trizol reagent (Invitrogen) following the manufacturer’s instructions. Next, cDNA synthesis was conducted with the PrimeScript^TM^ RT reagent Kit (Takara, Kusatsu, Japan), and cDNA amplification was performed using SYBR Premix Ex TaqTM II (Takara). *Gapdh* was chosen as a loading control. All the qRT-PCR primer sequences are listed in Supplementary Table S2.

### 4.12. Western Blotting

Proteins were extracted as previously described [20]. The protein concentration was measured using the BCA Assay Kit following the manufacturer’s protocol (Thermo Fisher Scientific, Waltham, MA, USA). Protein samples were resolved by SDS-PAGE gel electrophoresis, transferred to PVDF membranes (Bio-Rad, Hercules, CA, USA), and blocked with 5% BSA, followed by overnight treatment with primary antibodies at 4 °C. After washing with TBST buffer, membranes were treated with secondary antibodies for 1 h at room temperature. Signals on immunoblots were examined using the ODYSSEY Imaging System (LI-COR), and data were quantified with ImageJ software (Version 1.49).

### 4.13. Statistical Analysis

Data are expressed as mean ± SEM. A Shapiro–Wilk test was initially used to determine the normality of data, and all data in this article passed the normality distribution test. Comparisons between 2 groups were analyzed with a 2-tailed Student’s *t*-test. Differences between multiple groups were performed using a one-way ANOVA followed by Tukey’s post hoc tests. A *p*-value less than 0.05 described a statistically significant difference. All statistical analyses were performed using Prism software (GraphPad, CA, USA, Version 8.0).

## 5. Conclusions

For the first time, this study demonstrated that EG ethanolic extract attenuates EC-mediated inflammation and inhibits cardiac inflammatory responses, thereby alleviating cardiac I/R injury. A combined effect on the NF-κB and MAPK inflammatory signaling pathways is responsible for EG extracts’ anti-endothelial inflammatory and cardioprotective properties. Our findings shed light on the clinical application of EG ethanolic extract in coping with I/R-induced inflammation and the resulting cardiac injury.

## Figures and Tables

**Table 1:** Identification of the main components in EG extract based on ESI-MS.

No.	Compound	Formula	Molecular Mass	Charge Form	*m*/*z*	Major MS/MS Fragments	Peak Intensity	Relative Ratio (%)
1	Ethyllinolenate	C_20_H_34_O_2_	306	[M + H]^+^	307	123.59, 185.16, 221.2, 233.07, 261.16, 277.17, 289.14	2677	6.46
2	Tridecanoic acid	C_13_H_26_O_2_	214	[M + H]^+^	215	159.06	3910	9.44
3	Pentadecanoic acid	C_15_H_30_O_2_	242	[M + H − H_2_O]^+^	225	95.04, 169.1, 181.17, 193.09, 197.22, 207.09	3886	9.38
4	Kaempferol	C_15_H_10_O_6_	286	[M + H]^+^	287	213.21, 231.1, 241.1, 269.15	3490	8.42
5	Astragalin	C_21_H_20_O_11_	448	[M + N_a_]^+^	471	185.1, 309.04, 453.22	4327	10.44
6	Apigenin	C_15_H_10_O_5_	270	[M + H]^+^	271	145.2, 197.09, 215.17, 243.06, 253.25	3139	7.58
7	Daidzein	C_15_H_10_O_4_	254	[M + H]^+^	255	137.8, 199.11, 227.23	5841	14.10
8	Pro-lle	C_11_H_20_N_2_O_3_	228	[M + H − H_2_O]^+^	211	126.09, 183.11, 194.33	4888	11.80
9	GIn-Asp	C_9_H_15_N_3_O_6_	261	[M + H − H_2_O]^+^	244	180.09, 226.27, 235.43	5993	14.46
10	Cys(Trioxidation)-Pro	C_8_H_14_N_2_O_6_S	267	[M + H]^+^	268	222.11, 250.3	4783	11.54
11	GLu-His	C_11_H_16_N_4_O_5_	284	[M + H]^+^	285	249.26, 257.33, 267.07	2454	5.92
12	Daphnoretin	C_19_H_12_O_7_	352	[M + H]^+^	353	164.02, 179.02, 338.11	41,433	100.00
13	Dihydrokaempferol	C_15_H_12_O_6_	288	[M + H]^+^	289	195.31, 243.15, 271.29	4031	9.73
14	dl-arginine	C_6_H_14_N_4_O_2_	174	[M + H]^+^	175	60.13, 116.08, 130.16, 158.13	11,556	27.89
15	Palmital	C_16_H_32_O	240	[M + H]^+^	241	197.19, 211.25, 223.15,	2562	6.18
16	Adenosine	C_10_H_13_N_5_O_4_	267	[M + H]^+^	268	85.01, 136.14, 178.12	4783	11.54
17	Xanthene-9-carboxylic acid	C_14_H_10_O_3_	226	[M + H]^+^	227	100.92, 155.05, 181.23, 209.14, 217.59	7490	18.08
18	Mevalonic acid	C_6_H_12_O_4_	148	[M + H]^+^	149	121.01,	2280	5.50
19	1,4-Benzenediol, 2-methyl-	C_7_H_8_O_2_	124	[M + H]^+^	125	81.1, 107.04	2321	5.60
20	Sebacic acid	C_10_H_18_O_4_	202	[M + H − H_2_O]^+^	185	139.05, 149.24, 157.08, 167.02	31,738	76.60
21	Jasmonic acid	C_12_H_18_O_3_	210	[M + H − H_2_O]^+^	193	157.28, 175.15	5934	14.32
22	Salicylic acid	C_7_H_6_O_3_	138	[M + H]^+^	139	69.19, 111.05, 121.19	6156	14.86
23	Dimethyl phthalate	C_10_H_10_O_4_	194	[M + H − CH4O]^+^	163	119.21, 133.01	3348	8.08
24	Hexadecanoic acid	C_16_H_32_O_2_	256	[M − H]^−^	255	227.25, 237.25	37,768	97.08
25	Kaempferol 3-*O*-rutinoside	C_27_H_30_O_15_	594	[M − H]^−^	593	229.16, 257.15, 285.05, 327.14, 357.21	38,902	100.00
26	Femlic acid	C_10_H_10_O_4_	194	[M − H]^−^	193	134.12, 149.05	626	1.61
27	9*S*,11*R*,15*S*-trihydroxy-5*Z*-prostanoic acid	C_20_H_36_O_5_	356	[M − H]^−^	355	193.06, 201.11, 293.34, 311.18, 319.2, 337.11	2144	5.51
28	3-Hydroxy-3-methylglutaric acid	C_6_H_10_O_5_	162	[M − H]^−^	161	57.07, 99.03, 117.11, 143.17	3936	10.12

Note: The relative ratio indicates the signal intensity of the compound as a percentage of the peak intensity of the 12th compound (Daphnoretin, positive ionization mode) or the 25th compound (Kaempferol 3-*O*-rutinoside, negative ionization mode).

## Data Availability

The datasets supporting the conclusions of this study will be available from the corresponding authors upon reasonable request. The data are not publicly available due to privacy or ethical restrictions.

## Supplementary Materials

The following supporting information can be downloaded at: https://www.mdpi.com/article/10.3390/molecules29051068/s1, Table S1: Chemicals and reagents used in this study; Table S2: Sequences of qRT-PCR primers used in this study; Figure S1: The total ion chromatogram of EG extract by ESI-MS; Figure S2: The chemical structures of identified compounds in EG extract.
