# Supplementary material for: Edgeworthia gardneri (Wall.) Meisn. Ethanolic Extract Attenuates Endothelial Activation and Alleviates Cardiac Ischemia-Reperfusion Injury
Source: Molecules. 2024 Feb 29;29(5):1068. doi: 10.3390/molecules29051068 (PMC10935089; doi:10.3390/molecules29051068)
Supplement: Supplementary file 1 [file molecules-29-01068-s001.zip › molecules-2841678-supplementary.pdf]

Edgeworthia gardneri (Wall.) Meisn. Ethanolic Extract Attenuates Endothelial Activation and Alleviates Cardiac Ischemia-Reperfusion Injury

Xiaoya Lang [1,†], Chao Zhong [1,2,*,†], Lingqing Su [1], Manman Qin [1], Yanfei Xie [1], Dan Shan [2], Yaru Cui [1,2], Min Shi [1], Min Li [3], Hexiu Quan [1], Liang Qiu [1], Guoyue Zhong [3] and Jun Yu [2,*]

[1] Center for Translational Medicine, Jiangxi University of Chinese Medicine, Nanchang 330004, China;
langxiaoya@jxutcm.edu.cn (X.L.); 13606525622@163.com (L.S.); 20142016@jxutcm.edu.cn (M.Q.);
20070945@jxutcm.edu.cn (Y.X.); cuiyaru@jxutcm.edu.cn (Y.C.); 20010381@jxutcm.edu.cn (M.S.); 20101063@jxutcm.edu.cn (H.Q.); 20091057@jxutcm.edu.cn (L.Q.)

[2] Department of Cardiovascular Sciences, Center for Metabolic Disease Research, Lewis Katz School of
Medicine, Temple University, Philadelphia, PA 19140, USA; tuq93597@temple.edu

[3] Center for Traditional Chinese Medicine Resources and Ethnic Medicine, Jiangxi University of Chinese Medicine, Nanchang 330004, China; 20131004@jxutcm.edu.cn (M.L.); 20111025@jxutcm.edu.cn (G.Z.)

* Correspondence: zhongchao21@163.com (C.Z.); jun.yu@temple.edu (J.Y.)

† These authors contributed equally to this work.

Supplementary Table S1: Chemicals and reagents used in this study

Chemicals/reagents	Cat number	Company
Ethanol	64-17-5	Xilong Science
Dimethyl sulfoxide (DMSO)	D8370	Solarbio
2,3,5-triphenyl tetrazolium chloride (TTC)	T8170	Solarbio
10% Neutral Buffered Formalin Fixative	G2162	Solarbio
Hematoxylin	51275	Sigma-Aldrich
Eosin	E4009	Sigma-Aldrich
MTT	M5655	Sigma-Aldrich
DAB Kit	CW2069S	Cwbio
Trizol	15596-018	Invitrogen
PrimeScriptTM RT Reagent Kit	RR047A	Takara
SYBR Premix Ex TaqTM II	RR820A	Takara
ECM culture medium	1001-b, 0025, 1052, and 0503	ScienCell
RIPA buffer	KGP703-100	KeyGen
Protease inhibitors	E312-01	Vazyme
Tumor necrosis factor-α (TNF-α)	300-01A	PeproTech

BCA Protein Assay Kit	23225	Thermo Fisher Scientific
Anti-p65	6956	Cell Signaling Technology
Anti-p-p65	3033	Cell Signaling Technology
Anti-p38 MAPK	8690	Cell Signaling Technology
Anti-p-p38 MAPK	9216	Cell Signaling Technology
Anti-JNK	9252	Cell Signaling Technology
Anti-p-JNK	9255	Cell Signaling Technology
Anti-ERK	4696	Cell Signaling Technology
Anti-p-ERK	9101	Cell Signaling Technology
Anti-CD68	ab125212	Abcam
IRDye® 800CW Donkey anti-rabbit IgG	926-32213	LI-COR
IRDye® 680RD Donkey anti-mouse IgG	926-68072	LI-COR
Alexa Fluor 594 Goat anti-Mouse IgG (H+L)	A-11005	Invitrogen
Horseradish peroxidase-conjugated goat anti-rabbit IgG (H+L)	SE134	Solarbio
BAY11-7082	B5556	Sigma-Aldrich
SP600125	S5567	Sigma-Aldrich
PD98059	P215	Sigma-Aldrich

SB203580	S8307	Sigma-Aldrich

Supplementary Table S2: Sequences of qRT-PCR primers used in this study

Species	Genes	Primer sequences (5'-3')
Rat	*Tnf-α*	Forward: GTGATCGGTCCCAACAAGGA<br>Reverse: CTTGGTGGTTTGCTACGACG
	Il-1β	Forward: TGACTCGTGGGATGATGACG<br>Reverse: CAGCTCACATGGGTCAGACA
	Il-6	Forward: CCAGTTGCCTTCTTGGGACT<br>Reverse: CTGGTCTGTTGTGGGTGGTA
	Icam-1	Forward: ACCACGGAGCCAATTTCTCA<br>Reverse: TCGAGCTTCAGGACCCTAGT
	Gapdh	Forward: ACAGCAACAGGGTGGTGGAC<br>Reverse: TTTGAGGGTGCAGCGAACTT
Human	*TNF-α*	Forward: CCTCTCTCTAATCAGCCCTCTG<br>Reverse: GAGGACCTGGGAGTAGATGAG
	IL-6	Forward: ACTCACCTCTTCAGAACGAATTG<br>Reverse: CCATCTTTGGAAGGTTCAGGTTG
	MCP-1	Forward: TGTCCCAAAGAAGCTGTGATC<br>Reverse: ATTCTTGGGTTGTGGAGTGAG
	VCAM-1	Forward: TTTGACAGGCTGGAGATAGACT<br>Reverse: TCAATGTGTAATTTAGCTCGGCA
	ICAM-1	Forward: TTGGGCATAGAGACCCCGTT<br>Reverse: GCACATTGCTCAGTTCATACACC
	GAPDH	Forward: AGAAGGCTGGGGCTCATTTG<br>Reverse: AGGGGCCATCCACAGTCTTC

Supplementary Figure S1

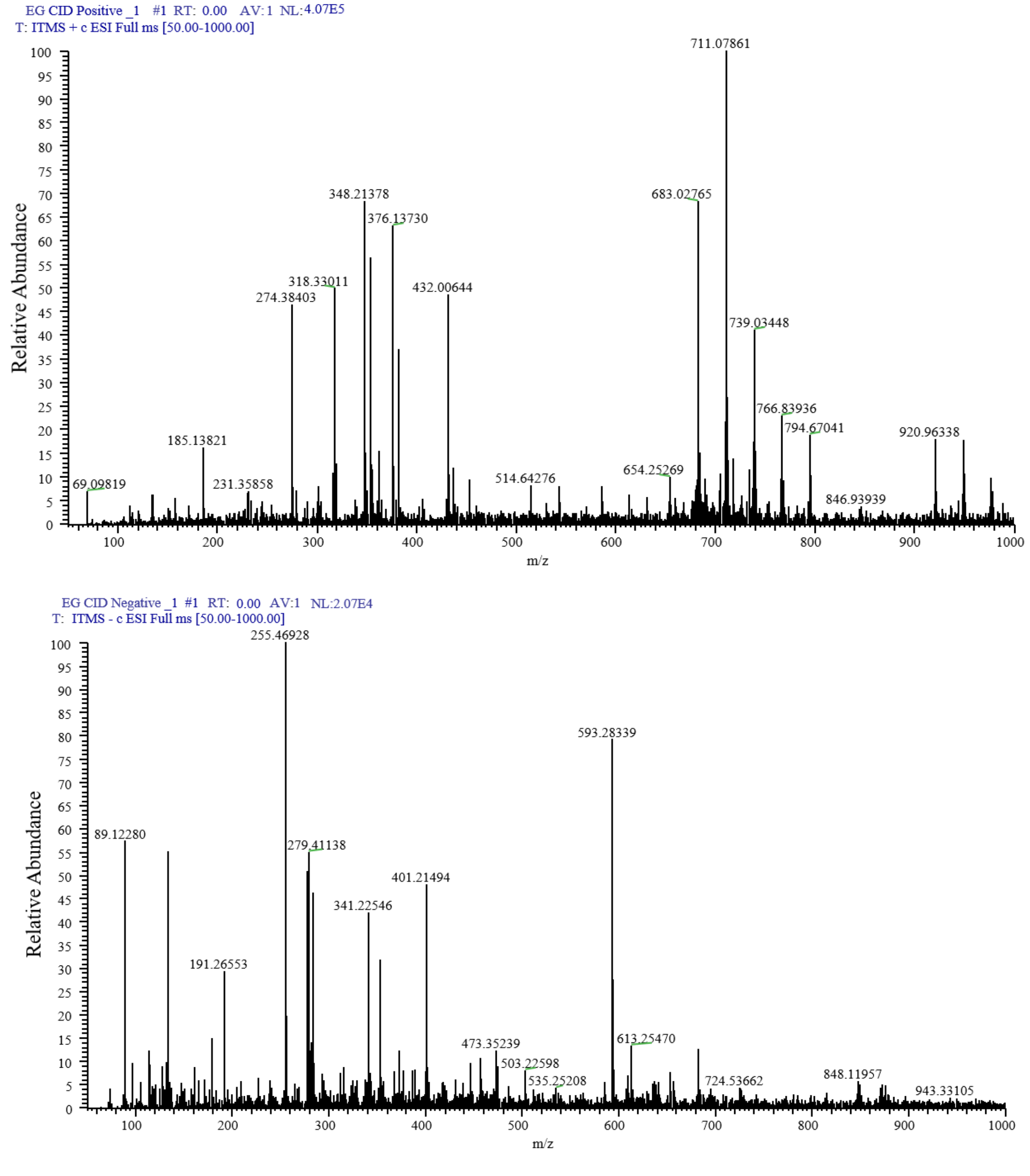

Figure S1. The total ion chromatogram of EG extract by ESI-MS. Upper: positive ionization mode; Lower: negative ionization mode.

Supplementary Figure S2

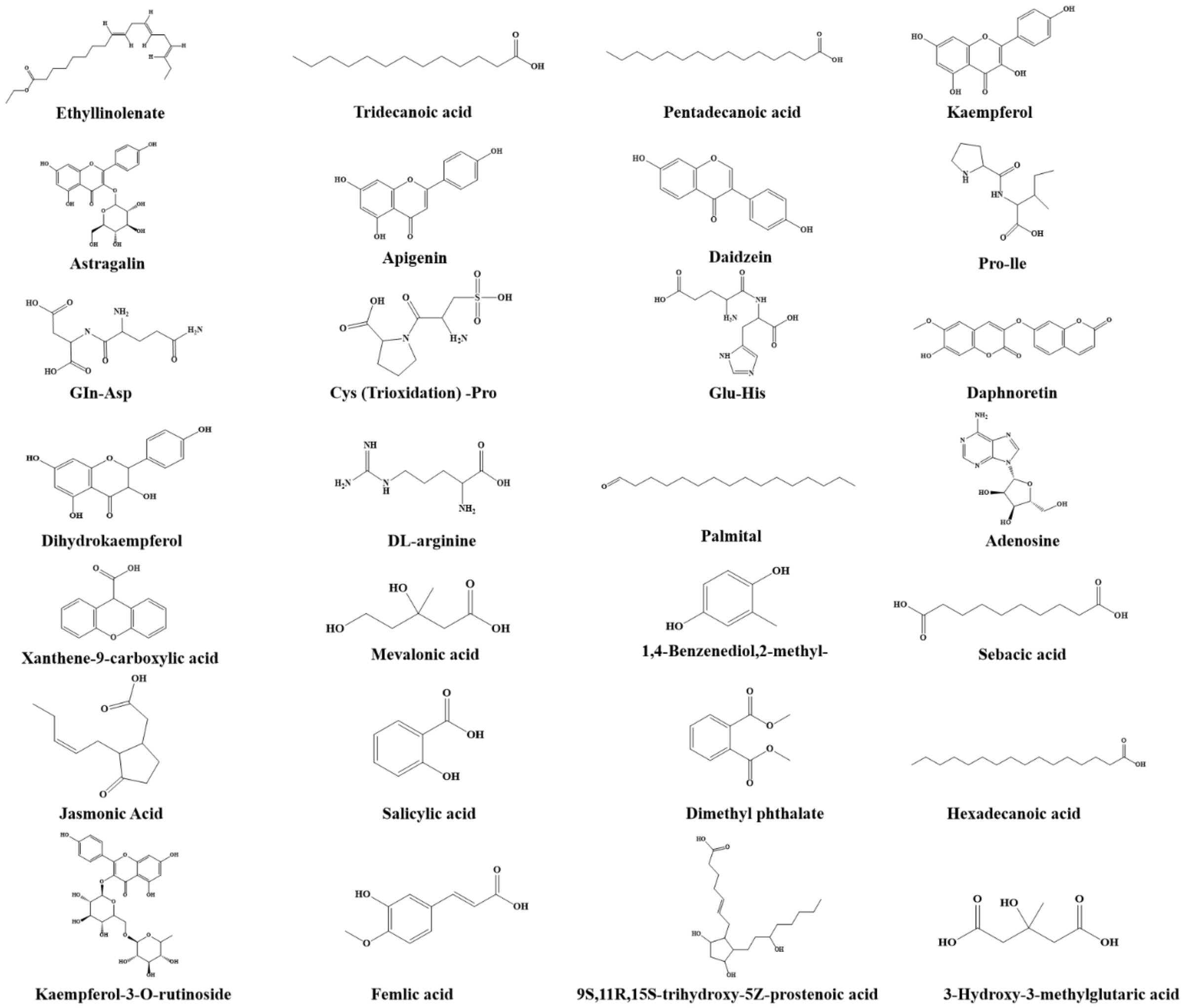

Figure S2. The chemical structures of identified compounds in EG extract.
